# Immunoinformatics Design of Multi-Epitope Peptide-Based Vaccine Against *Schistosoma mansoni* Using Transmembrane Proteins as a Target

**DOI:** 10.3389/fimmu.2021.621706

**Published:** 2021-03-02

**Authors:** Rodrigo C. O. Sanches, Sandeep Tiwari, Laís C. G. Ferreira, Flávio M. Oliveira, Marcelo D. Lopes, Maria J. F. Passos, Eduardo H. B. Maia, Alex G. Taranto, Rodrigo Kato, Vasco A. C. Azevedo, Debora O. Lopes

**Affiliations:** ^1^Laboratório de Biologia Molecular, Universidade Federal de São João del-Rei, Divinópolis, Brazil; ^2^Programa de Pós-Graduação em Bioinformática, Instituto de Ciências Biológicas, Universidade Federal de Minas Gerais (UFMG), Belo Horizonte, Brazil; ^3^Laboratório de Química Farmacêutica Medicinal, Universidade Federal de São João del-Rei, Divinópolis, Brazil; ^4^Centro Federal de Educação Tecnológica de Minas Gerais (CEFET-MG), Divinópolis, Brazil

**Keywords:** schistosomiasis, immunoinformatics, multi-epitope vaccine, chimeric antigen, bioinformatics, transmembrane proteins

## Abstract

Schistosomiasis remains a serious health issue nowadays for an estimated one billion people in 79 countries around the world. Great efforts have been made to identify good vaccine candidates during the last decades, but only three molecules reached clinical trials so far. The reverse vaccinology approach has become an attractive option for vaccine design, especially regarding parasites like *Schistosoma* spp. that present limitations for culture maintenance. This strategy also has prompted the construction of multi-epitope based vaccines, with great immunological foreseen properties as well as being less prone to contamination, autoimmunity, and allergenic responses. Therefore, in this study we applied a robust immunoinformatics approach, targeting *S. mansoni* transmembrane proteins, in order to construct a chimeric antigen. Initially, the search for all hypothetical transmembrane proteins in GeneDB provided a total of 584 sequences. Using the PSORT II and CCTOP servers we reduced this to 37 plasma membrane proteins, from which extracellular domains were used for epitope prediction. Nineteen common MHC-I and MHC-II binding epitopes, from eight proteins, comprised the final multi-epitope construct, along with suitable adjuvants. The final chimeric multi-epitope vaccine was predicted as prone to induce B-cell and IFN-γ based immunity, as well as presented itself as stable and non-allergenic molecule. Finally, molecular docking and molecular dynamics foresee stable interactions between the putative antigen and the immune receptor TLR 4. Our results indicate that the multi-epitope vaccine might stimulate humoral and cellular immune responses and could be a potential vaccine candidate against schistosomiasis.

## Introduction

Schistosomiasis is a human parasitic disease caused by trematode parasites in the genus *Schistosoma*. The World Health Organization (WHO) classifies schistosomiasis as the second most importance socioeconomic disease in the world and as the third most relevant parasitic disease regarding public health (1). Worldwide it is estimated that there are 700 million people at risk of infection and more than 230 million infected (2, 3). The most relevant species for human health are: *Schistosoma mansoni, Schistosoma japonicum*, and *Schistosoma haematobium*. Global areas affected by these species include: Africa and the Middle East (*S. haematobium*); Africa and South America (*S. mansoni*); China and the Philippines (*S. japonicum*) (4).

Strategies for schistosomiasis control and elimination have been implemented and reexamined in the last three decades (5–8). Major efforts consist of diagnosis and preventive chemotherapy using the drug Praziquantel, which is primarily administered to school-age children between 5 and 15 years of age (9, 10). A recent study demonstrated that the integration of treatment and diagnosis with: health education, control of snails (intermediate host), supply of clean water, and sanitation has a great positive impact (11). Nevertheless, it is worth noting that Praziquantel presents little efficacy against juvenile forms of the parasite and since it is the only medicine used for mass treatment, raises legitimate concerns about drug resistance (4).

The aforementioned data emphasize the relevance of an integrated disease combating program harboring distinct actions. Therefore, vaccination would certainly have a great impact on disease elimination agendas, acting as an essential complementary tool (12, 13). Nonetheless, *Schistosoma* has biological features that turn it into a tough task. Different parasite evolutionary phases and their remarkable ability to evade and subvert immunological mechanisms of elimination represent a significant bottleneck (14). The existence of different causative species also highlights the relevance of a pan-approach in the development of vaccines for the disease. First vaccine trials were carried out using the attenuated parasite (cercaria). Despite good protection results, this vaccination approach was not used in humans due to safety concerns (15, 16). Subsequent studies revealed several parasite antigenic molecules with potential to become a vaccine, such as: Sm14 (17), Sm28GST (18), Sh28GST (19), TSP-2 (20), Smp-80 (21), and Sm29 (22). Currently, some of these antigens are in clinical phase studies like Sm14^1^, Sh28GST (23) and TSP-2^2^, yet crucial steps are required until they become licensed.

The development of vaccines against multicellular parasites, like *Schistosoma*, naturally presents more challenges when compared to unicellular organisms, mainly due to their physiological complexity (24) and the difficulty of culture maintenance. Besides, schistosomiasis is classed as a Neglected Tropical Disease (NTD), for which financial funding is historically constrained (25). Nevertheless, the development of bioinformatics in recent decades has contributed to improving this scenario. Predictive software has been used to screen pathogens genetic information in order to find potential vaccine targets (26). Besides improving cost, time and safety issues, compared to traditional methods, the immunoinformatics approach has also driven the development of multi-epitope or chimeric vaccines. Different studies have applied it to construct potentially immunogenic molecules from viruses (27), bacteria (28), and parasites (29) antigens. Multi-epitope vaccines allow the induction of a broad immune response since it might harbor B-cell and T-cell epitopes. Moreover, this strategy also may enhance immunogenicity and long-lasting immune responses by coupling adjuvants to the whole sequence (30).

Generally, molecules exposed on the surface of microorganisms, such as plasma membrane proteins are most likely to interact with the host's immune system, in addition to performing essential functions for pathogen homeostasis (31). This is especially true for extracellular parasites that can't enter the cell and are less prone to directly trigger intracellular immune receptors, essentially relying on exposed (or secreted) molecules for its immune activation (32). The most exposed region of *Schistosoma* adult parasites consists of a syncytial layer, named tegument. This structure is connected to underlying nucleated cell bodies, from where vesicles are released reaching the apical tegument membrane. Such vesicles aid to cover parasite with a membranocalyx, which is essential for immune attack evasion, displaying a vital function (33). Therefore, plasma membrane proteins found in the tegument has been investigated over the years and some of them proved to be promising vaccine candidates, like TSP-2 and Sm29 (34).

Given the above, here we based our work on an immunoinformatics approach to identify all hypothetical *S. mansoni* plasma membrane proteins and, from their extracellular domains, to predict epitopes in order to build a multi-epitope based antigen. Suitable adjuvants and linkers were introduced during the antigen assembly. Antigenicity, physicochemical and structural properties were evaluated for the putative chimeric antigen, as well as its interaction with an immune receptor (TLR 4).

## Methods

### Retrieval of *Schistosoma mansoni* Proteins and Preliminary Analysis

Proteins sequences were obtained from the GeneDB database^3^. All hypothetical transmembrane proteins from *S. mansoni* available in this database were gathered. The PSORT II server^4^ was used in order to identify and select only transmembrane sequences most likely to being located in the plasma membrane (35).

In order to confirm PSORT II prediction and identify the extracellular domains from plasma membrane sequences, CCTOP topology predictor^5^ was used. This server presents a consensus-constrained method of prediction, based on the hidden Markov Model, and considers on its algorithm ten other topology prediction methods (36). Those extracellular domains presenting at least 30 amino-acids length were selected for further analysis.

### Cytotoxic T Lymphocyte (CTL) and Helper T Lymphocyte (HTL) Epitope Prediction

The aforementioned extracellular domains were submitted to IEDB^6^ server to predict CTLs^7^ and HTLs^8^ epitopes, applying the IEDB recommended 2020.04 (NetMHCpan EL 4.0) and IEDB recommended 2.22 prediction methods, respectively. Epitopes were predicted based on HLA allele frequencies and reference sets with maximal population coverage, provided by the server. For CLTs, the dataset included 108 alleles (37) and for HTLs it comprised 27 alleles (38). The chosen MHC class I alleles automatically limit available peptides lengths. Epitopes with percentile rank <0.5 were selected. On the other hand, 12–18-mer length epitopes were predicted for the MHC class II allele reference set and those presenting either percentile rank <0.5 and IC_50_ value <500 nM were selected for further analysis.

### B-Cell Epitopes Prediction

Proteins extracellular domains were also used to predict linear B-cell epitopes, which was carried out by ABCpred server^9^. This software uses the Recurrent Neural Network (RNN) method, trained on a data set of non-redundant B-cell epitopes and non-epitopes from Bcipep and Swiss-Prot databases, respectively (39). Applying server standard threshold (0.51), 20-mer length epitopes were selected for next steps.

### Selecting Overlapped Epitopes

Initially, CLTs and B-cell epitopes, provided by the same extracellular region, were aligned. CTLs fitting within B-cell sequences were then submitted to the analysis of immunogenicity. The chosen tool^10^ is hosted by IEDB and uses amino acid properties as well as their position within the peptide to predict the immunogenicity of a peptide-MHC complex (40). Epitopes presenting positive scores were selected.

Same alignment approach was conducted for HTLs and B-cell epitopes. Only HTLs fitting within B-cell sequences and presenting IC_50_ < 50 nM were selected. Subsequently, CTLs and HTLs epitopes overlapped with B-cell sequences were aligned with each other to select those matching. For all these alignments the MULTALIN tool was used^11^.

### Epitopes Antigenicity

**Figure 1:**
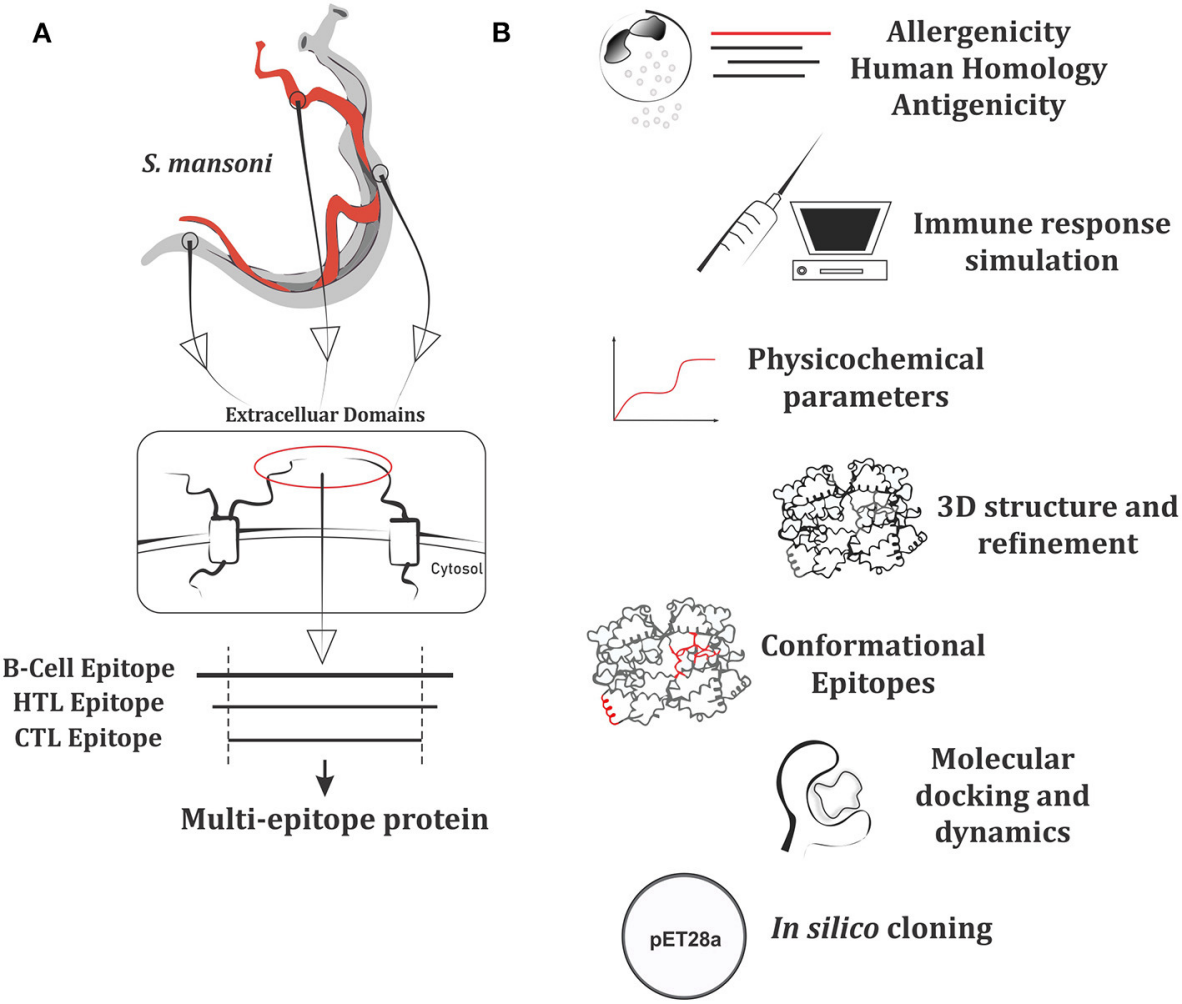
Workflow summarizing the approach of multi-epitope protein construction and subsequent analysis. **(A)** Putative antigen conception made from epitopes of plasma membrane extracellular domains. **(B)** Set of analysis performed for the multi-epitope protein.

The ability of previously selected epitopes to act as an antigen was evaluated by the VaxiJen server^12^. The server prediction method does not rely on alignment or homology. Instead, it predicts protective peptides based on autocross-covariance (ACC) transformation of protein sequences using uniform vectors of the main amino acids physicochemical properties (41). For parasite model, the server provides a threshold of 0.5. Out of the final epitopes, only those with antigenicity score above 0.7 were chosen as components of the chimeric antigen. A summary of epitopes prediction and selection steps are shown in Figure 1.

### Construction of Multi-Epitope Vaccine Sequence

In order to fuse the selected epitopes in a rationally immunogenic fashion we used suitable linkers and adjuvants. The well-described linkers EAAAK, GGGS, GPGPG, HEYGAEALERAG, and AAY were used to connect different components. The N-terminal region of the multi-epitope protein starts with HIV TAT peptide that plays a cell penetrating role. Next, to improve the immunogenicity, we choose the 50S ribosomal protein L7/L12 (Locus RL7_MYCTU) (Accession no. P9WHE3). Subsequent, the Pan DR epitope (PADRE – AKFVAAWTLKAAA) adjuvant was also fused to act as a T helper Lymphocyte stimulus. Then, CTLs epitopes were added followed by HTLs. Finally, the C-terminal portion included a 6xHis tag for further purification assays.

### Homology Analysis

To evaluate similarity with human proteins and therefore reduce autoimmunity possibilities, a BLAST^13^ was carried out. The whole multi-epitope vaccine sequence and its epitopes individually were submitted against UniProtKB Human database. Moreover, similarity with the most relevant *Schistosoma* species (*S. japonicum* and *S. haematobium*) were evaluated using UniProt Knowledgebase dataset. This former analysis was conducted for the final eight proteins from which we have predicted our epitopes.

Considering the importance of the microbiota in maintaining the organism's homeostasis, we submited the multi-epitope protein against the proteome of commonly found gut microbiota, with the aid of Pipeline Builder for Identification of drug Targets (PBIT) server^14^. PBIT is an online server which is widely used for screening of pathogen proteomes in terms of some critical features that must be fulfilled for a protein to serve as potential drug/vaccine target for humans (42). Proteins presenting sequence identity <50% with gut microflora proteome and having an e-value cutoff > 0.005 were considered as non-homologous.

### Antigenicity and Allergenicity

To assess the antigenicity of whole multi-epitope vaccine, VaxiJen server^15^ was used once more considering the threshold for parasite model (0.5). In addition, to evaluate allergenic potential, the multi-epitope protein was submitted to AllerTop v.2.0 server^16^. The method of this server is based on ACC transformation of protein sequences into uniform equal-length vectors. Proteins are classified based on training set containing 2427 known allergens from different species and 2427 non-allergens (43).

### IFN-γ Inducing Epitope Prediction

The presence of IFN-γ inducing epitopes in the multi-epitope vaccine sequence were predicted by IFNepitope server^17^ using the scan module. Among algorithms models available, the motif and support vector machine (SVM) hybrid model was selected. The server generates all possible overlapping peptides from antigen, based on either IFN-γ inducing and IFN-γ non-inducing datasets (44). Prediction score >1.0 was set as a threshold value for epitope selection.

Considering that IFNepitope server was trained with MHC II epitopes, we further submitted all final MHC II epitopes selected before chimera assembly. This analysis was carried out in order to check whether the chimeric-MHC II epitopes we selected would be predicted as an IFN- γ inducing peptide. In this case, the server module used was the module “Predict,” which ranks the inserted peptides in positive or negative order, based on their potential to induce IFN- γ.

### Proteasomal Cleavage/TAP Transport/MHC Class I Combined Prediction

After multi-epitope vaccine conception, we decided to confirm if upon processing steps in the cell, our chimeric-MHC I epitopes would be generated. In order to perform this we applied the Proteasomal cleavage/TAP transport/MHC class I combined predictor^18^ (45), found in IEDB server^6^. The chosen method was the IEDB recommended and the proteasome type was the immunoproteasome. Epitopes were predicted based on HLA allele set mentioned earlier, in the CTL epitope prediction section.

### Immune Simulation for Vaccine Efficacy

To further characterize the immunogenicity and the immune response of the multi-epitope vaccine, *in silico* immune simulations were conducted by C-ImmSim server^19^. C-ImmSim is an agent-based model that uses a position-specific scoring matrix (PSSM) for immune epitope forecast and machine learning techniques for prediction of immune interactions. The server's prediction contemplates simulation of three distinct anatomical regions related to immune responses in mammals: the bone marrow, the thymus and a tertiary lymphatic organ, like lymph node, tonsil and spleen (46). Considering the immunization schedule for schistosomiasis vaccines under clinical trials, such as rSh28GST (23) and rSm14 (47), we adopted three doses at intervals of 4 weeks. Therefore, injections containing 1,000 vaccine proteins each were administered four weeks apart at 1, 84, and 168 time-steps (each time-step is equivalent to 8 h in real-life and time-step 1 is injection at time = 0) with total 1,050 simulation steps (48). The remaining simulation parameters were kept defaults.

### Physicochemical Properties Analysis

A set of physicochemical parameters were assessed for the multi-epitope protein using ProtParam online server^20^. These parameters include amino acid and atomic composition, molecular weight, theoretical pI, aliphatic index, extinction coefficient, estimated half-life for three model organisms (*Escherichia coli*, yeast, and mammal cells) and the instability index (49). In turn the solubility was evaluated by SOLpro server^21^, which uses SVM model to predict the propensity of a protein to be soluble upon overexpression in *E. coli* and gives its corresponding probability (50).

### Tertiary Structure Prediction

Multi-epitope vaccine three-dimensional (3D) structure was obtained using the Iterative Threading Assembly Refinement (I-TASSER) server^22^. This server is an integrated platform for automated protein structure and function prediction based on the sequence-to-structure-to-function paradigm. Starting from an amino acid sequence, I-TASSER first generates three-dimensional (3D) atomic models from multiple threading alignments and iterative structural assembly simulations. An estimate accuracy of the predictions is provided based on the confidence score (C-score) of the modeling. C-score is typically in the range between−5 and 2, wherein a higher score reflects a model of better quality. In general, models with C-score > −1.5 have a correct fold (51).

### Refinement and Validation of the 3D Modeled Structure

The predicted vaccine 3D structure from I-TASSER was submitted to refinement in order to improve structure quality, both locally and globally. This task was carried out by GalaxyRefine server^23^. After sequence input, the server applies dynamics stimulation in order to perform structure perturbations and relaxation. Initially, for the first model, the structure perturbation is applied only to clusters of side-chains. Then, for second to fifth models, more aggressive perturbations to secondary structure elements and loops are also applied (52). In order to validate the refined tertiary structure, RAMPAGE web server^24^ was used. The main output of this server is a Ramachandran plot, which permits to visualize the percentage of residues in favored, allowed and disallowed regions, based on dihedral angles phi (ϕ) and psi (ψ) of each amino acid in the protein (53).

### Discontinuous B-Cell Epitope Prediction

Discontinuous or conformational B-cell epitopes are formed as a result of protein folding that can bring into proximity distant residues and form it. It has been estimated that >90% of B-cell epitopes are discontinuous (54). In order to evaluate the presence of these epitopes, the refined and validated multi-epitope 3D structure was submitted to ElliPro server^25^.

### Molecular Docking of Vaccine Construct With TLR 4 Immune Receptor

The ability to trigger innate immune receptors is broadly described for *Schistosoma* antigens. Then, for docking analysis, the TLR 4 receptor structure was retrieved from the RCSB PDB database (PDB ID: 2Z63) and edited by Chimera software^26^, in order to remove attached oligosaccharides.

To predict the 3D structure of multi-epitope vaccine and TLR 4 receptor complex, the SwarmDock server was used. The server has a flexible protein-protein docking algorithm, which initially pre-process and minimize input structures and performs docking through a hybrid particle swarm optimization/local search. Next, it minimizes, re-ranks and clusters docked poses. Lastly, democratic clustered structures are provided as PDB format (55). The analysis was carried out by chosen full blind mode. In order to visualize hydrogen bonds and hydrophobic interactions between ligand (multi-epitope antigen) and receptor (TLR 4) the software LIGPLOT v2.2 was applied.

### Molecular Dynamics Simulation of the Receptor-Ligand Complex

Molecular dynamics (MD) simulation is applied to determine the stability of receptor-ligand complexes (48). Gromacs v5.0.5 (56) was used to study the structural properties and interaction between ligand (multi-epitope vaccine) and receptor (TLR 4) at the atomic level. All molecular dynamic simulation was performed using GROMOS96a force field. To guarantee that the geometry of the system is adequate and there are no steric clashes using the steepest descent algorithm approach, energy minimization was performed prior to simulation. During equilibration phase (100 ps), the temperature was increased up to 300 K and pressure up to 1 bar. Finally, the trajectories generated from the simulation (20 ns) were analyzed for the stability of the complex in terms of the root mean square deviation (RMSD) and root mean square fluctuation (RMSF) of backbone atoms relative.

### *In silico* Cloning

Finally, in order to provide a feasible plasmid construction harboring the multi-epitope sequence, Java Codon Adaptation Tool (JCat) server was used. This server provides a codon optimized version of the interest DNA sequence, based on a chosen organism (57). Here we choose *E. coli* (strain K12). The output also includes two more parameters; Codon Adaptation Index (CAI) and percentage of GC content. For CAI, the ideal value is 1.0 and the GC content should gather values between 30 and 70%. To carry the cloning into an expression vector, pET28a(+), SnapGene software was used. *SalI* and *BamHI* restriction sites were introduced flanking the multi-epitope sequence to ensure the suitable insertion into the plasmid.

## Results

### Selected T Lymphocyte Epitopes

Gene DB search provided 584 hypothetical transmembrane protein sequences, from which 37 were predicted as most likely located at plasma membrane by PSORT II server. Out of it, CCTOP predictor allowed us to identify 54 extracellular domains ≥ 30 amino acids.

IEDB server predicted promiscuous CTLs epitopes covering 108 alleles. It was found 13,981 epitopes presenting percentile rank <0.5, for all extracellular domains. In turn IEDB foreseen 6,893 HTLs epitopes presenting percentile rank <0.5 and IC_50_ < 500 nM, considering a set of 27 alleles. Diverse epitopes were predicted to more than one MHC alleles and they were considered as one in further analyses.

### Linear B-Cell Epitopes

The ABCpred server predicted linear B-cell epitopes for all 54 extracellular domains. The analysis generated a total of 1,583 sequences.

### Overlapping of Cellular and Humoral Inducing Epitopes

The alignment of multiple sequences aid to identify and select epitopes harboring cellular and humoral inducing ability. The first alignment was carried out between CTLs and B-cell epitopes, which generated a set of CTLs epitopes overlapping with B-cell sequences. Out of this, a second set of peptides were obtained through class I immunogenicity analysis. The outcome was a group of best CTL epitopes.

Similarly, a set of HTLs epitopes overlapping with B-cell sequences was obtained and improved by selecting only those with IC_50_ < 50 nM. Finally, both best epitopes groups (CTLs and HTLs) were aligned to each other and yielded 55 matched sequences.

### Final Candidates and Multi-Epitope Vaccine Conception

The best 55 epitopes were submitted to VaxiJen antigenicity analysis. Out of the total, 19 epitopes presented score >0.7 (Supplementary Table 1) and therefore were chosen as final components of the chimeric vaccine (Table 1).

**Table 1:** Final selected CTL and HTL epitopes overlapping with each other and with B-cell epitopes.

**Protein Code**	**CTL epitope**	**HTL epitope**	**B-Cell epitope**
Smp_127680	**ISPEEWFIF**	**ISPEEWFIF**AQSSILSCL	**ISPEEWFIFAQSSILSCL**LE
	**QSAAIIAAT**	RGN**QSAAIIAAT**NP	AAAALF**RGNQSAAIIAATNP**
Smp_128250	**ALLILSNWK**	TEQ**ALLILSNWK**LDP	PNFQF**TEQALLILSNWKLDP**
	**DYEQFTTSI**	QLDF**DYEQFTTSI**	GN**QLDFDYEQFTTSI**GSLST
Smp_145110	**APFIIMSHIF**	WL**APFIIMSHIF**S	RCHLSVD**WLAPFIIMSHIFS**
Smp_160120	**GVIGAGPYAI**		**GVIGAGPYAI**SQGWRWLTLY
	**SGVIGAGPY**		AAIIALT**SGVIGAGPY**AISQ
Smp_175510	**LYNFRFLLF**	HLSDAVQ**LYNFRFLLF**QG	**HLSDAVQLYNFRFLLFQG**TK
	**QLYNFRFLL**		HLSDAV**QLYNFRFLL**FQGTK
Smp_179660	**HINAFINRNW**	A**HINAFINRNW**PAIVTMA	VD**AHINAFINRNWPAIVTMA**
Smp_205910	**TTAVLAAAA**		ASS**TTAVLAAAA**AAGYIAQH
Smp_244490	**NTNPIIESII**		FLISN**NTNPIIESII**SRLSV

*The overlapped regions are in bold*.

**Figure 2:**
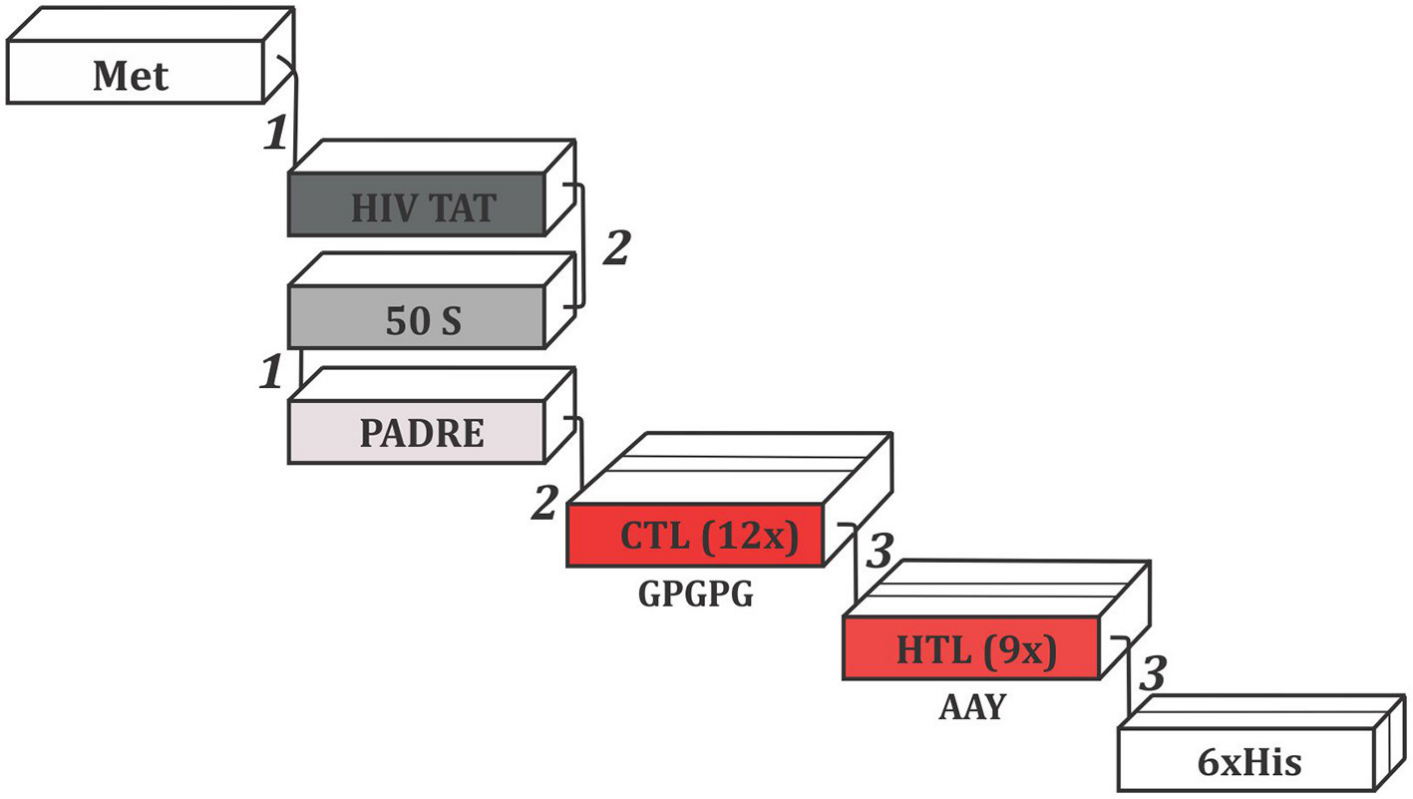
Schematic representation of the multi-epitope vaccine construct. Epitopes were connected by the following linkers: (1) EAAAK, (2) GGGS, and (3) HEYGAEALERAG. The GPGPG and AAY linkers, showed in the figure, were used to merge CTLs and HTL epitopes, respectively. Met, Methionine; HIV TAT, Cell penetrating peptide; PADRE, Pan DR epitope; 50S, 50S ribosomal protein L7/L12; CTL, Cytotoxic T Lymphocyte epitope; HTL, Humoral T Lymphocyte epitope; His, Histidine.

In order to construct the multi-epitope vaccine, we have used suitable adjuvants and linkers. The final sequence is represented in Figure 2. N-terminal portion starts with a methionine connected to the HIV TAT peptide by EAAAK linker. Next, a GGGS linker was used to add the 50S ribosomal protein L7/L12, which establish its connection with PADRE adjuvant via EAAAK linker. Between PADRE and the first CTL epitope, GGGS was inserted once more. A total of twelve CTL epitopes were attached using GPGPG linkers. The last CTL epitope connects to the first HTL epitope through HEYGAEALERAG linker. The connection of all seven HTL epitopes were assisted by AAY linker. Finally, the C-terminal portion included a 6 × His tail attached via HEYGAEALERAG linker.

### The Multi-Epitope Vaccine Gathers Prerequisites of a Safety and Effective Antigen

The search for similarity between multi-epitope vaccine and *Homo sapiens* proteins did not reveal any match. The same result was observed when chimeric epitopes were individually submitted to a BLAST analysis. As expected, the search for similarity between multi-epitope protein and gut microbiome revealed considerable identity for the 50S ribosomal protein L7/L12, but non-homology for other components, considering server's cutoff (58, 59) (Supplementary Table 2). Thereby, the constituents of our chimeric molecule kept unaltered. Moreover, based on allergens and non-allergens sets, AllerTOP predicted the multi-epitope vaccine as non-allergen. These results suggest that our hypothetical antigen might be safety in *in vivo* assays.

In addition to check epitopes antigenicity before vaccine construction, we also evaluated this parameter for the multi-epitope vaccine itself. The VaxiJen server provided a score value of 0.5145, above of the threshold established for parasite model (41), classifying our molecule as a probable antigen. Moreover, a considerable amount of INF-γ inducing epitopes were identified in the multi-epitope vaccine sequence. Out of the total 490 epitopes (15-mer) predicted, 76 presented score >1 and therefore were selected (Supplementary Table 3). Still regarding IFNepitope server, we applied an additional analysis to all MHC II epitopes selected before chimera conception and found that among final seven chimeric-HTLs epitopes, 4 were predicted as IFN-γ inducing epitopes (Supplementary Table 4).

**Figure 3:**
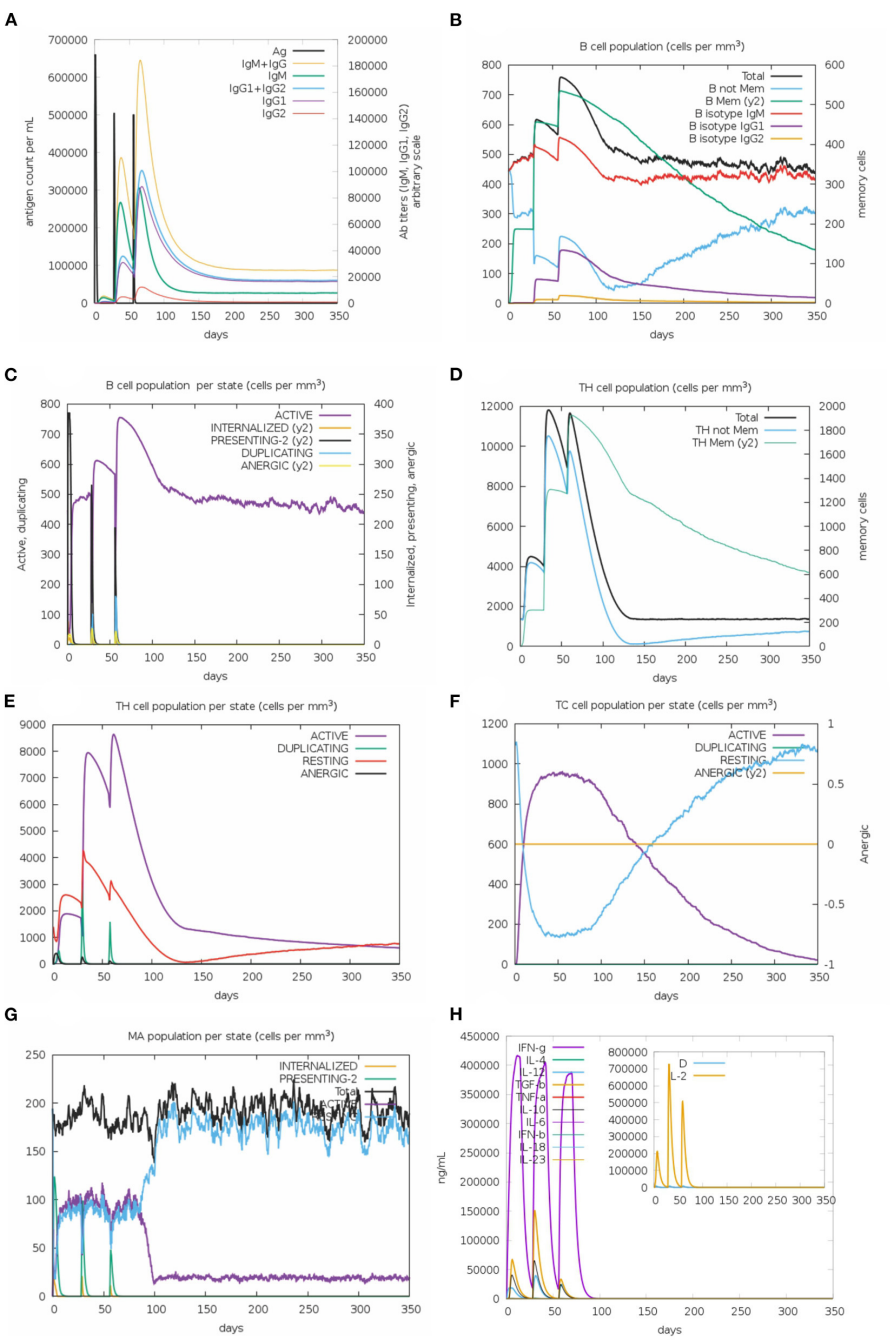
*In silico* simulation of immune response for the multi-epitope vaccine. **(A)** Antigen and immunoglobulins, **(B)** B-cell population per state, **(C)** B-cell population, **(D)** T helper cell population per state, **(E)** T helper cell population, **(F)** T cytotoxic cell population per state, **(G)** Macrophage population per state, and **(H)** production of cytokine and interleukins.

Regarding the results of *in silico* immune response, the behavior was generally consistent with actual outcomes induced upon vaccination; secondary and tertiary responses significantly higher than the primary one. Elevated titers of IgM + IgG, IgM, IgG1 + IgG2, and IgG1 antibodies were observed, followed by reduction in antigen concentration (Figure 3A). Accordingly, an increase of active B-cell population was observed and memory B-cell was especially elevated (Figures 3B,C). Similar behavior was detected for T helper (Th) and T cytotoxic cells (Figures 3D–F). Among innate immune cells, the results demonstrated increased macrophage activity (Figure 3G). Lastly, levels of IFN-γ and IL-2 were highly evident (Figure 3H). These data highlight the potential of multi-epitope vaccine in inducing long-lasting humoral and cellular immune responses.

### Physicochemical Analysis Reveals Encouraging Parameters for Vaccine Manufacture

Based on amino acid sequence, ProtParam predicted a set of physicochemical parameters for the multi-epitope vaccine. It has foreseen a size of 52 kDa and isoeletric point (pI) of 5.93. The total numbers of positive and negative charged residues were 38 and 46, respectively. The instability index (II) was computed to be 31.24, which classifies the multi-epitope vaccine as a stable molecule. This stability was reinforced by the aliphatic index, 86.06. The higher the aliphatic index, the more stable is a protein in a broad range of temperatures. During heterologous expression in bacteria or yeast is essential a long period of half-life. In this sense, our molecule presented an estimated half-life of 30 h in mammalian reticulocytes (*in vitro*), >20 h in yeast and >10 h in *E. coli*, both *in vivo*. Regarding solubility, SOLpro server provided good prediction (0.9010) upon overexpression in *E. coli*.

### The Multi-Epitope Vaccine Adopts a Favored 3D Structure

The I-TASSER server starts modeling process from structure templates identified in the PDB library. The server can generate tens of thousands of template alignments, but it only uses the ones of the highest significance, which is measured by the *Z*-score (>1 mean good alignment). Ten best templates (*Z*-score ranging from 1.01 to 5.59) were used to predict five potential tertiary structures of the multi-epitope vaccine. Each of these five models presented an individual *C*-score value: −0.41, −2.34, −2.59, −4.30 and −2.70. *C*-score range is typically between −5 and 2, with values >-1.5 indicating a correct global topology. Therefore, we selected the *C*-score −0.41 model as our multi-epitope tertiary structure.

**Figure 4:**
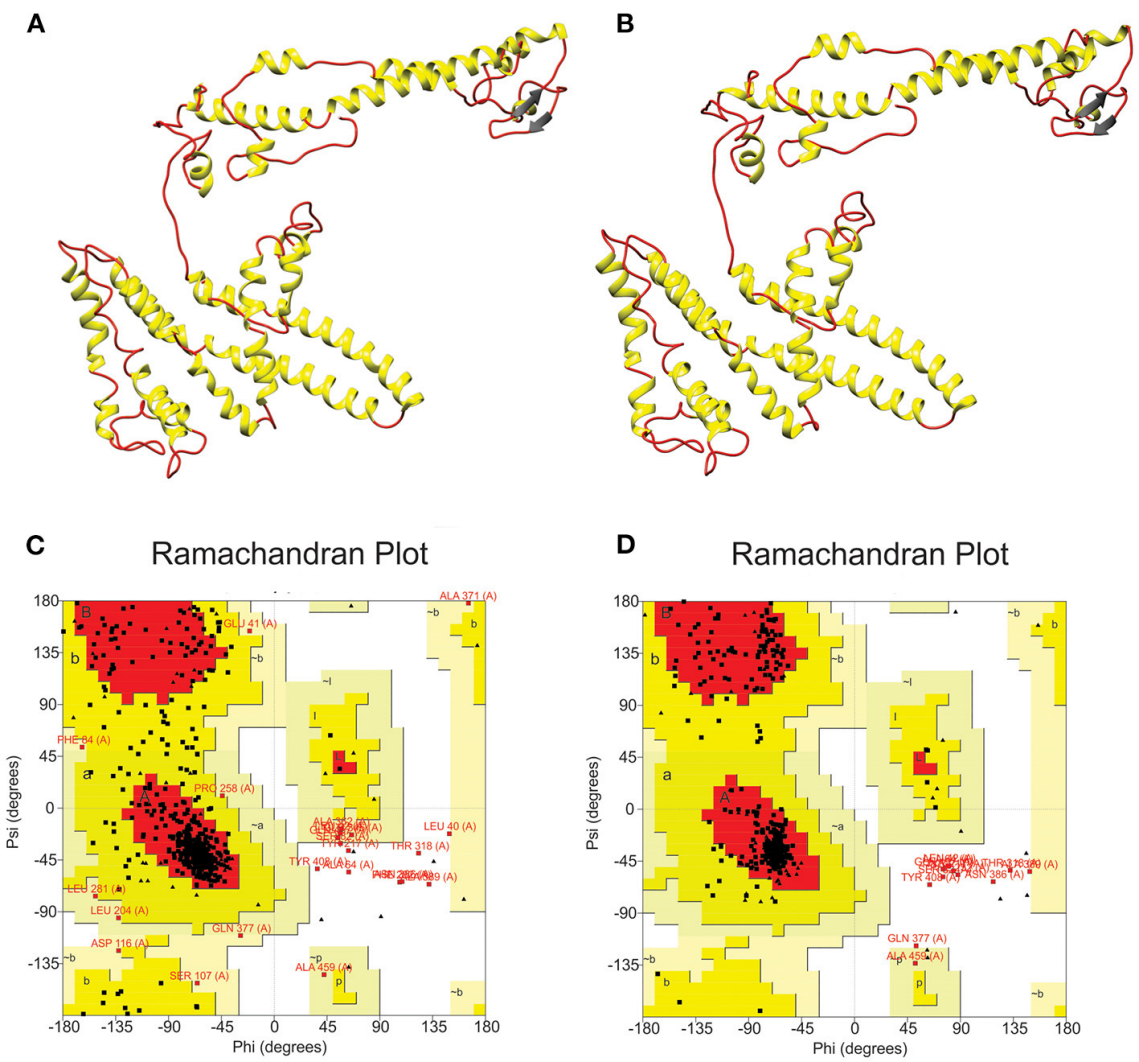
Multi-epitope vaccine modeling and refinement. **(A)** Tertiary structure generated by I-TASSER server presenting **(C)** 76.4% of the residues in favored regions, 18% in allowed regions and 2.3% in disallowed regions. **(B)** Refined tertiary structure obtained by GalaxyRefine server presenting **(D)** 90.2, 6.8, and 2.8% of the residues in favored, allowed, and disallowed regions, respectively.

After choosing the best 3D model, a refinement process was carried out by GalaxyWeb server in order to improve structure quality. As a result, the server generated five refined models (Supplementary Table 5). To select the best 3D model, we compared initial and refined models considering the Ramachandran plot, which was generated by RAMPAGE server. The comparison established the initial model presented 76.4% of the residues in favored regions, 18% in allowed regions and 2.3% in disallowed regions (Figures 4A,B). On the other hand, after the refinement, model harbored 90.2, 6.8, and 2.8% of the residues in favored, allowed and disallowed regions, respectively (Figures 4C,D).

### The Putative Vaccine Folding Generates Conformational B-Cell Epitopes

According to ElliPro server, a total of 253 residues were distributed in 10 conformational B-cell epitopes, with scores ranging from 0.60 to 0.877. The epitopes size ranged from four to 77 residues (Supplementary Table 6).

### TLR 4 Receptor Establishes Interactions With Multi-Epitope Vaccine

**Figure 5:**
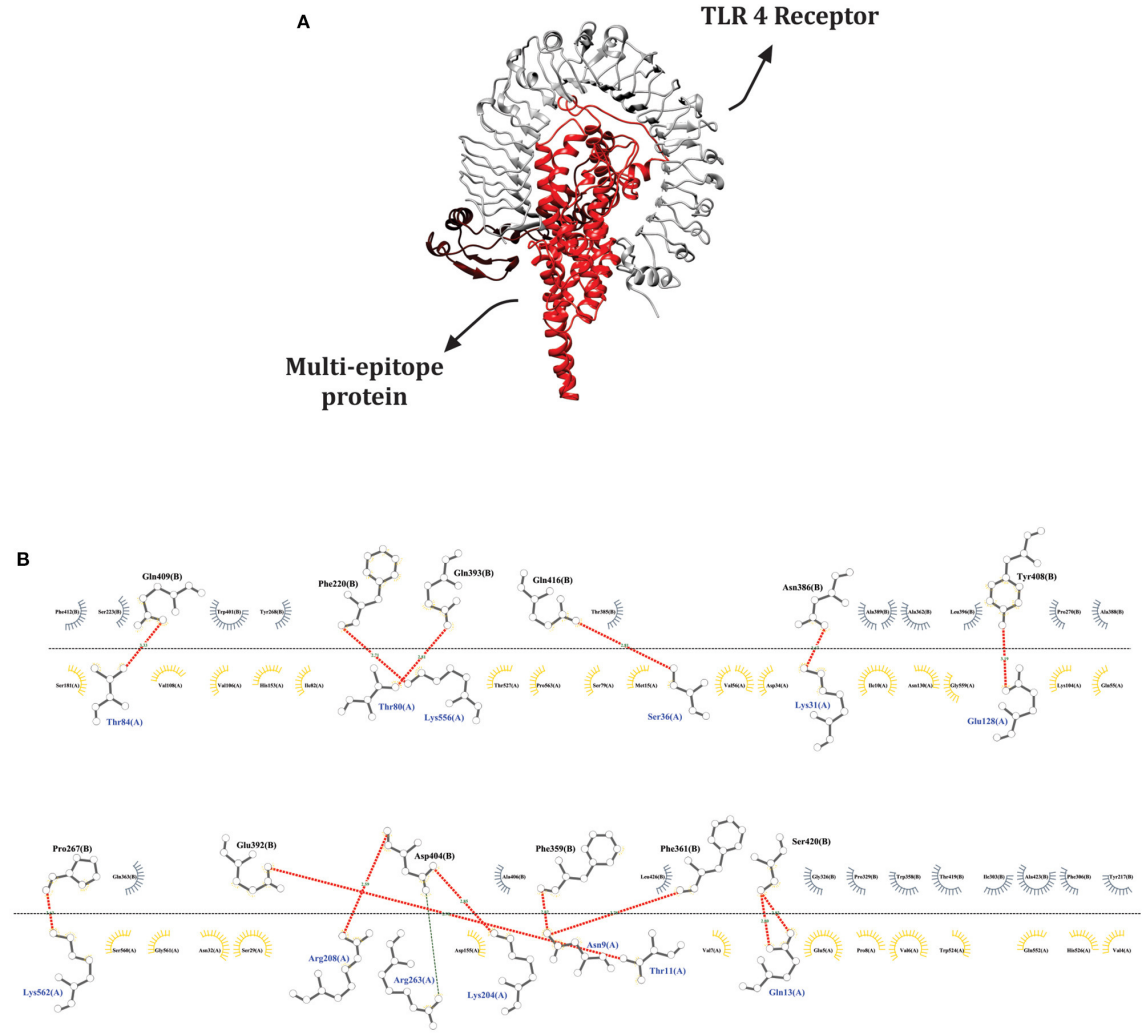
Ligand-receptor docked complex using the Swarmdock server. **(A)** Docked structure visualization generated by Chimera software; TLR 4 (receptor) in gray and the multi-epitope protein (ligand) in red. **(B)** Interactions between ligand and receptor provided by the LIGPLOT v.2.2 software. Red lines represent hydrogen bonds, gray semi-circles denote hydrophobic interactions made by the ligand and yellow semi-circles represent hydrophobic interactions made by the receptor.

The SwarmDock server and LIGPROT v2.2 software allowed us simulate and analyze possible interactions between the ligand (multi-epitope vaccine) and the immune receptor (TLR 4). The best docked structure was selected based on minimum energy value, −43.71. This complex exhibited 14 hydrogen bonds, 29 hydrophobic interactions for the ligand and 21 for the receptor, as demonstrated in Figure 5. These data suggest that our chimeric vaccine could stimulate an important immune receptor, for this reason a dynamic simulation was further conducted.

### Molecular Dynamics Predicts a Stable Ligand-Receptor Interaction

**Figure 6:**
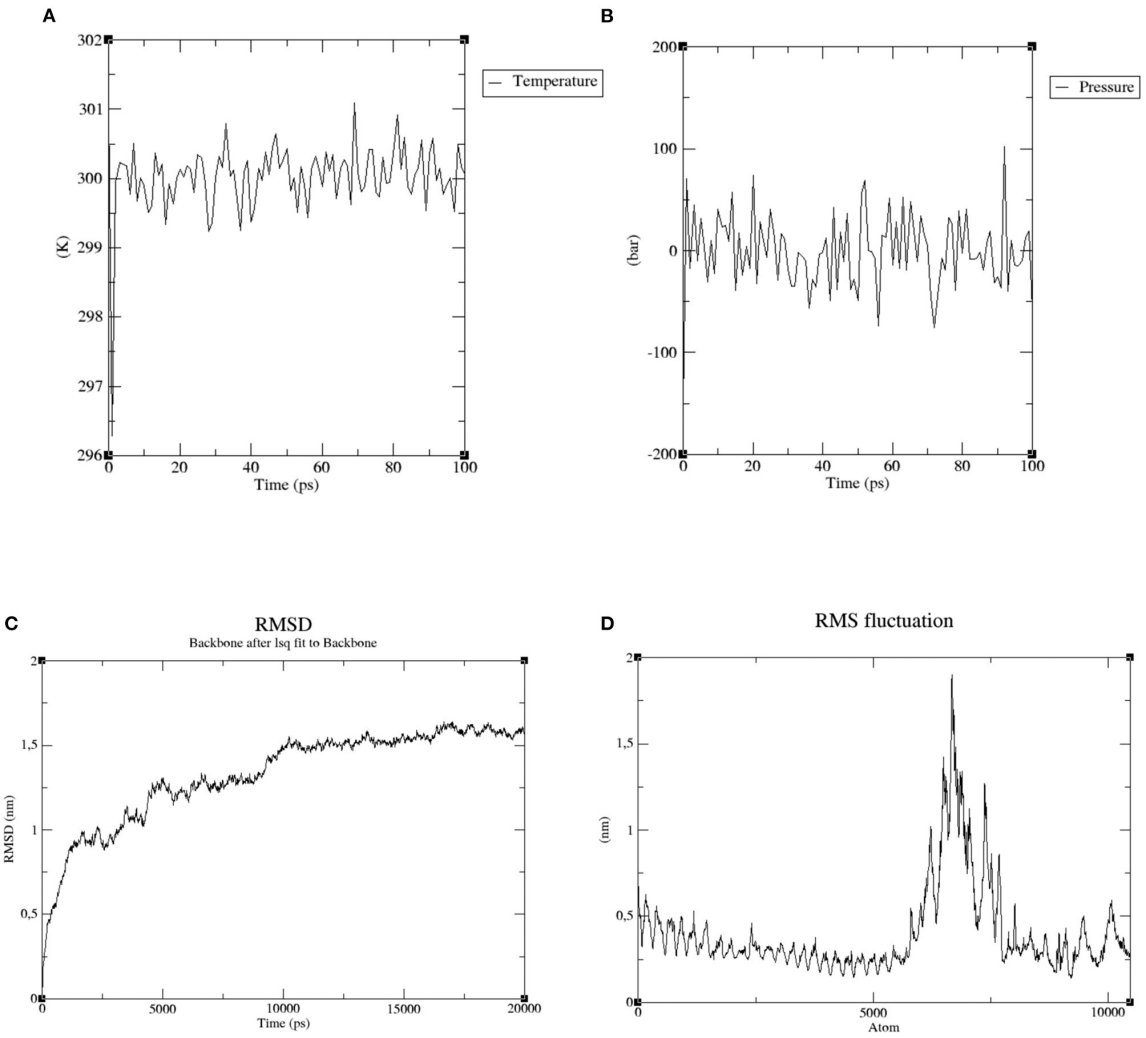
Molecular dynamics simulation of the ligand-receptor complex. **(A)** Temperature (Kelvin) fluctuation of the ligand-receptor complex during the equilibration phase (100 ps). **(B)** The ligand-receptor pressure plot, measured over the equilibration phase (100 ps). **(C)** RMSD—Root Mean Square Deviation of the ligand-receptor complex presenting no substantial displacement, indicating stable atomic interaction between the two molecules. **(D)** RMSF—Root Mean Square Fluctuation plot of the ligand-receptor complex, representing the flexibility of the amino acids side chain.

In order to confirm the proper engagement of TLR 4 with the putative multi-epitope vaccine, a MD simulation was conducted. After energy minimization step, temperature and pressure equilibration phase were assessed during 100 ps. Temperature raised quickly and reached 300 K, which was kept throughout the interval analyzed (Figure 6A). Similarly, pressure plot demonstrated a fluctuation in pressure around 0.25 bar over the entire equilibration phase (Figure 6B).

Subsequently, the final trajectory was used to analyze some essential parameters; the first one was the RMSD. This parameter measures reflect the stability between the receptor and ligand where mild fluctuations point to a stable interaction. Figure 6C shows a RMSD plot ranging from 0.25 nm to 1.5 nm, after 20 ns of time interval, which is considered as a mild fluctuation.

The second parameter, RMSF, represents amino acids side chains fluctuations. Elevated fluctuations at the plot indicate highly flexible regions in the receptor-ligand complex while mild ones point to continuous interaction between receptor and ligand molecules. Most of our selected complex has presented uninterrupted interactions (0.5 nm), except for a specific region of highly flexibility (1.8 nm), as demonstrated in Figure 6D.

### *In silico* Cloning

**Figure 7:**
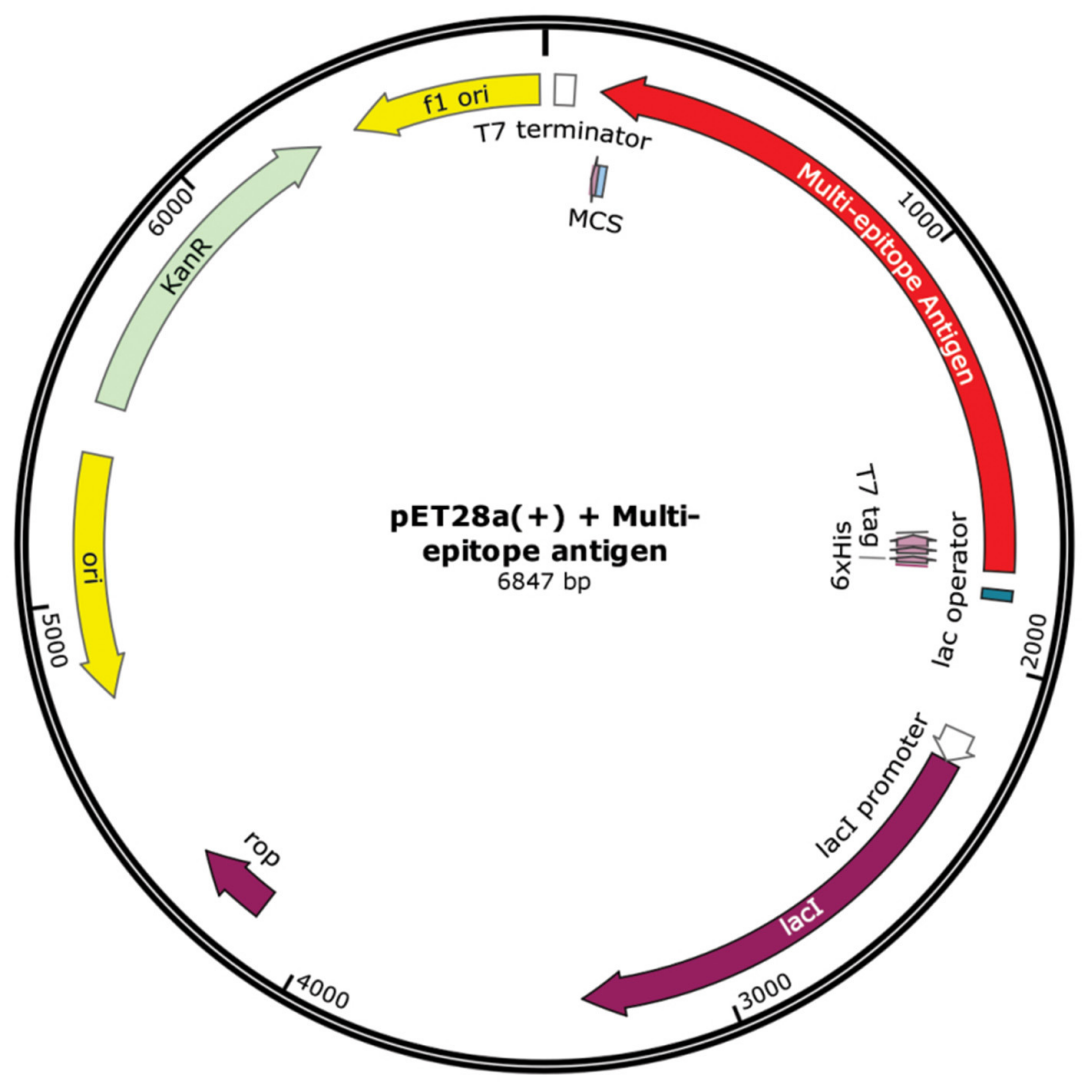
*In silico* cloning. Multi-epitope vaccine sequence cloned into pET28a (+) expression vector, represented in red color. The insert was added between *SalI* and *BamHI* restriction sites.

Jcat server generated an optimized DNA sequence presenting good parameters. The CAI value was 1.0, indicating high chance of increased expression. GC content reached 54.25%, which remains in the optimal range (30–70%). Expression vector pET 28a (+) carrying the multi-epitope vaccine insert is represented in the Figure 7.

## Discussion

Historically, vaccine development has been considered the best cost-effective method for combating diseases caused by infectious pathogens (60). For decades, researchers have been working to find an effective antigen and an appropriate formulation for schistosomiasis vaccine. Such effort allowed identification of several promising antigens and the development of a consensus view on the importance of vaccination within combating disease programs (61). A wide range of approaches have been addressed; from attenuated parasite to DNA vaccines, also including recombinant molecules, synthetic peptides, and chimeric proteins (12). Chimeric or multivalent antigens, in particular, represent an attractive strategy given the possibility of linking potentially immunogenic molecules in a single structure. Almost two decades ago, the first studies related to this approach in the context of schistosomiasis were published (62, 63). Since then, technologies such as genomics and bioinformatics have evolved, improving the process of building chimeric antigens. Screening of epitopes considering a great diversity of HLAs, structural investigation of the vaccine candidate, predictions of antigenicity and evaluation of interactions with human receptors are some of the possibilities raised by the progression of computational analysis (64). This immunoinformatics approach is very advantageous due to its safety, time, and cost-effectiveness. Therefore, in recent years, several studies have been applying these methods for organisms like viruses, bacteria, and parasites, yielding promising molecules (27–29).

Recently, Rahmani et al. (65) reported the construction of a potential vaccine candidate against schistosomiasis based on a multi-epitope approach followed by a robust *in silico* analysis. As constituents of this putative antigen, authors selected T and B-cell epitopes found in seven major vaccine targets (Sm14, Sm21.7, Sm23, Sm29, Smp80, Sm-CB, and SM-TSP-2) described for the disease (65). Using a similar workflow, we have proposed here the construction of a new multi-epitope vaccine candidate, considering a distinct protein source.

Naturally, pathogens' surface proteins are prone to interact with the host's immune system, and therefore, they may induce an immune response (31). However, in multicellular organisms such as *S. mansoni* the most exposed proteins are found in a specific apical syncytial layer, the tegument. Since there is no database that provides specific tegument's protein sequences and considering the potential of parasite's proteins not yet studied, we started our approach by analyzing all hypothetical transmembrane sequences, 584, provided by the GeneDB database. Subsequently, the usage of PSORT II and CCTOP servers allowed to filter 37 plasma membrane proteins, of which 54 extracellular regions, with at least 30 amino acids, were obtained.

The extracellular domains aforementioned provided a large collection of epitopes, predicted based on a set of HLAs presenting wide population coverage (>97% for MHC class I and >99% for MHC class II). Our selection criteria allowed us to reach a final set of epitopes, CTLs and HTLs, with great affinity scores for MHC molecules, and being potentially recognized by B-cells. This feature is essential for vaccine approach, given the relevance of neutralizing antibodies against the pathogen and the support of helper T cells in the prolonged antibodies production, as well as in maintaining the activation of CTL response (66). Furthermore, the selected epitopes were all predicted as possible antigens, stressing the immunogenic potential of these sequences.

Connection between epitopes, as well as their merge with other chimera components, was achieved by using linkers. Studies of naturally-occurring multiple domain proteins have prompted the use of linker peptides for artificial protein fusion (67). In the context of multi-epitope vaccines, the main advantages of using linkers are the reduced probability of junctional antigens formation and their ability to improve antigen processing and presentation (68). Nevertheless, structural flexibility and rigidity are also relevant parameters modulated by linkers. Based on the study by Rahmani et al. (65) and on several other investigations regarding multi-epitope vaccine for pathogens (28, 29, 68) and cancer (69, 70), we have used the following linkers: EAAAK, GGGS, GPGPG, HEYGAEALERAG, and AAY. The choice of best distribution of the linkers throughout the chimera sequence was based on the stability of 3D structure. In other words, if the multi-epitope protein yielded an unstable structure, the linkers organization would be shifted. Given the functional role of the 50S ribosomal protein L7/L12, as a TLR 4 agonist (71), EAAAK was used to provide rigidity, reducing possible interference of other protein's regions in the interaction between adjuvant and its receptor. On the other hand, a flexibility contribution was provided by GGGS. The remaining linkers were used mainly considering their ability to induce HTL immune response (GPGPG) (72) and to act as cleavage sites for the proteasomal (AAY, HEYGAEALERAG) and lysosomal (HEYGAEALERAG) systems (73, 74). The usage of a proteasomal cleavage predictor in our study suggested that upon processing steps in the cell, our chimeric-MHC I epitopes would be generated (Supplementary Table 7). This result reinforces that chosen linkers and their distribution were suitable.

Recently, Cell Penetrating Peptides (CPPs) have been widely investigated as possible agents for vaccine delivery. The integration of these peptides with antigenic sequences aims to improve their uptake by Antigen Presenting Cells (APCs), thereby favoring antigen processing and presentation processes (75). Here, we have used the HIV TAT protein, the first CPP described and extensively explored (76). Investigations including different pathogens, such as Hepatitis B virus (77) and *Leishmania major* (78), clearly demonstrate the potential of TAT improving the immune response against the CPP-fusioned antigens when compared to non-fusioned.

Studies addressing immunologic mechanisms triggered by the most promising vaccine antigens for schistosomiasis have revealed CD4^+^ T cell responses, and IFN-γ production, as outcomes closely related to protection against the parasite infection (21, 79, 80). Considering these observations, the choice of 50S ribosomal protein L7/L12 and PADRE adjuvants, as additional components of the multi-epitope vaccine, appeared suitable. Lee et al. (71) demonstrated that 50S ribosomal protein L7/L12 has the ability to induce Dendritic cell (DC) maturation, which later are capable to activate naive T cells, resulting in CD4^+^ and CD8^+^ IFN-γ secreting cells. Similarly, Ghaffari-Nazari et al. (81) observed that presence of PADRE adjuvant in a vaccine formulation comprising multi-epitope protein and CpG-oligodeoxynucleotides (CpG-ODN) promoted an improvement in the anti-tumor (lobular carcinoma) immune response, by inducing expansion of CD4^+^ and CD8^+^ IFN-γ producing subpopulations. In our study the outcome of IFNepitope server analysis predicted 76 IFN-γ inducing epitopes in the structure of the multi-epitope protein. Besides, *in silico* immune response simulation predicted the secretion of high levels of IFN-γ and a long-lasting cellular response. In addition, C-ImmSim server foreseen a relevant antibody production after immunization (IgM + IgG, IgM, IgG1 + IgG2, and IgG1), which is also described as essential for combating *Schistosoma* infection (12). These data emphasize the possibility of our candidate to induce an effective immune response, able to protect against the disease.

The ability to manipulate host's immune response is a hallmark of helminths. They accomplish that by interacting with different Pattern Recognition Receptors (PRRs), primarily through their secretion and excretion products (32). Durães et al. (82) have demonstrated that the co-culture of Bone Marrow Derived Dendritic Cells (BMDCs) with schistosomula tegument (Smteg) can induce the expression of co-stimulatory molecules in these cells, as well as stimulated cytokines production, like IL-12p40 and TNF-α. Such cellular events took place in a TLR 4-dependent manner, suggesting interaction between parasite's antigens and this immune receptor (82). Here, we observed, through protein-protein docking, that our multi-epitope vaccine establishes several intermolecular interactions with human TLR 4, such as hydrogen bonds and hydrophobic contacts. Coherently, RMSD measures obtained by molecular dynamic simulation, has indicated stable interaction between the ligand and the receptor. Yet, even though parasite's antigens might be able to interact with TLRs, it is valuable to emphasize that our multi-epitope protein carries a TLR 4 agonist (50S ribosomal protein L7/L12) in its sequence, which indeed improves potential receptor interactions.

The physicochemical features foreseen for the multi-epitope candidate strongly suggest heterologous expression and antigen purification as viable processes. The predicted half-life in *E. coli* (10h) and the molecule stability (instability index 31.24 and aliphatic index 86.06) propose the usage of this organism as a platform for heterologous expression. For this reason, we have optimized vaccine candidate codons, based on *E. coli* strain K12, and performed the *in silico* cloning in a common expression vector, pET28a (+). Moreover, predicted isoelectric point (5.93) indicates that under neutral pH conditions the protein will assume a negative global charge, which lean toward to aid affinity purification procedure, as those carried in Nickel (Ni^2+^) immobilized columns (83, 84). Besides, substantial changes in the protein structure are not expected to occur under these pH conditions. Finally, solubility prediction (0.9010) also corroborates for a succeed manufacturing process.

After decades searching for an effective antigen to be used as proper vaccine against schistosomiasis, few molecules reached clinical trials, keeping the pursuit for alternative approaches open (61). An attractive feature for vaccine antigens is the conservation among pathogen's species, which can promote cross-protection. In our study, the final selected proteins which provided the epitopes for the chimeric putative antigen, presented a high percentage of identity among *S. japonicum* and *S. haematobium* (Supplementary Table 8). On the other hand, since we have chosen to work with hypothetical plasma membrane proteins, there is no annotated function available for them. However, out of the final eight proteins, two presented known superfamilies motifs when submitted to InterProScan (data not shown). WD-40 repeats were found in Smp_127680, these motifs act as a site for protein-protein or protein-DNA interaction, and proteins containing WD40 repeats are known to serve as platforms for the assembly of protein complexes or mediators of transient interplay among other proteins (85). Smp_175510 in turn presented an immunoglobulin-like domain, which is involved in a variety of functions, including cell-cell recognition, cell-surface receptors, and muscle structure (86).

In general, extracellular domains of proteins will always be an appreciable source of vaccine candidates for different pathogens. Likewise, studies of hypothetical proteins can translate into the discovery of potential candidates and stimulate their investigation/annotation. Therefore, we have combined these concepts into a multi-epitope protein that presented itself as a good putative antigen. Immunogenic, physicochemical and structural properties suggest that our vaccine candidate might yield promising results on near future *in vitro* and *in vivo* assays.

## Data Availability Statement

The original contributions presented in the study are included in the article/Supplementary Material, further inquiries can be directed to the corresponding author/s.

## Author Contributions

RS, ST, VA, and DL designed the whole analysis. RS, LF, FO, ML, MP, EM, AT, and RK carried out most of the analysis. RS, ST, and DL wrote the manuscript. RS prepared figures. DL submitted this paper. All authors reviewed the manuscript.

## Conflict of Interest

The authors declare that the research was conducted in the absence of any commercial or financial relationships that could be construed as a potential conflict of interest.

## Footnotes

^1^https://clinicaltrials.gov/ct2/show/NCT03041766

^2^https://clinicaltrials.gov/ct2/show/NCT03910972

^3^http://www.genedb.org/Homepage

^4^http://psort1.hgc.jp/form2.html

^5^http://cctop.enzim.ttk.mta.hu/

^6^https://www.iedb.org/

^7^http://tools.iedb.org/mhci/

^8^http://tools.iedb.org/mhcii/

^9^https://webs.iiitd.edu.in/raghava/abcpred/index.html

^10^http://tools.iedb.org/immunogenicity/

^11^https://npsa-prabi.ibcp.fr/cgi-bin/npsa_automat.pl?page=/NPSA/npsa_multalin.html

^12^http://www.ddg-pharmfac.net/vaxijen/VaxiJen/VaxiJen.html

^13^https://www.ebi.ac.uk/Tools/sss/ncbiblast/

^14^http://www.pbit.bicnirrh.res.in/

^15^http://www.ddg-pharmfac.net/vaxijen/VaxiJen/VaxiJen.html

^16^https://www.ddg-pharmfac.net/AllerTOP/index.html

^17^http://crdd.osdd.net/raghava/ifnepitope/index.php

^18^http://tools.iedb.org/processing/

^19^http://150.146.2.1/C-IMMSIM/index.php

^20^https://web.expasy.org/protparam/

^21^http://scratch.proteomics.ics.uci.edu/index.html

^22^https://zhanglab.ccmb.med.umich.edu/I-TASSER/

^23^http://galaxy.seoklab.org/cgi-bin/submit.cgi?type=REFINE

^24^https://servicesn.mbi.ucla.edu/SAVES/

^25^http://tools.iedb.org/ellipro/

^26^https://www.cgl.ucsf.edu/chimera
