# Supplementary material for: Immunoinformatics Design of Multi-Epitope Peptide-Based Vaccine Against Schistosoma mansoni Using Transmembrane Proteins as a Target
Source: Front Immunol. 2021 Mar 2;12:621706. doi: 10.3389/fimmu.2021.621706 (PMC7961083; doi:10.3389/fimmu.2021.621706)
Supplement: Supplementary file 1 [file Data_Sheet_1.docx]

**TABLE 1.** Final CTL and HTL epitopes antigenicity score predicted by VaxiJen server.

| **Protein Code** | **CTL epitope** | **Antigenicity** | | **HTL epitope** | **Antigenicity** | |
| --- | --- | --- | --- | --- | --- | --- |
| Smp_127680 | ISPEEWFIF | 1.1379 | ISPEEWFIFAQSSILSCL | | 0.8788 |  |
|  | QSAAIIAAT | 0.8019 | RGNQSAAIIAATNP | | 1.0708 |  |
| Smp_128250 | ALLILSNWK | 1.7559 | TEQALLILSNWKLDP | | 0.7407 |  |
|  | DYEQFTTSI | 1.3545 | QLDFDYEQFTTSI | | 0.9171 |  |
| Smp_145110 | APFIIMSHIF | 1.1947 | WLAPFIIMSHIFS | | 1.5428 |  |
| Smp_160120 | GVIGAGPYAI | 1.2183 |  | |  |  |
|  | SGVIGAGPY | 2.2678 |  | |  |  |
| Smp_175510 | LYNFRFLLF | 2.5923 | HLSDAVQLYNFRFLLFQG | | 0.9001 |  |
|  | QLYNFRFLL | 1.7074 |  | |  |  |
| Smp_179660 | HINAFINRNW | 1.3342 | AHINAFINRNWPAIVTMA | | 0.8385 |  |
| Smp_205910 | TTAVLAAAA | 0.7816 |  | |  |  |
| Smp_244490 | NTNPIIESII | 1.1136 |  | |  |  |

**TABLE 2.** Similarity of the multi-epitope protein with gut microbiome.

| **Protein** | **Organism** | **Score (Bits)** | **E-Value** | **% Identity** |
| --- | --- | --- | --- | --- |
|  |  |  |  |  |
| 50S ribosomal protein L7/L12 | *Bifidobacterium*  *dentium* | 122 (305) | 3e-032 | 65 |
| 50S ribosomal protein L7/L12 | *Bifidobacterium*  *breve* | 121 (304) | 4e-032 | 66 |
| 50S ribosomal protein L7/L12 | *Bifidobacterium*  *adolescentis* | 121 (303) | 6e-032 | 65 |
| 50S ribosomal protein L7/L12 | *Bifidobacterium*  *longum* | 120 (300) | 1e-031 | 65 |
| 50S ribosomal protein L7/L12 | *Eggerthella*  *lenta* | 119 (299) | 2e-031 | 64 |
| 50S ribosomal protein L7/L12 | *Collinsella*  *aerofaciens* | 119 (298) | 3e-031 | 62 |
| 50S ribosomal protein L7/L12 | *Bifidobacterium*  *angulatum* | 118 (296) | 5e-031 | 65 |
| 50S ribosomal protein L7/L12 | *Collinsella*  *stercoris* | 118 (295) | 7e-031 | 59 |
| 50S ribosomal protein L7/L12 | *Collinsella*  *intestinalis* | 117 (293) | 1e-030 | 62 |
| 50S ribosomal protein L7/L12 | *Clostridium*  *hiranonis* | 114 (285) | 2e-029 | 54 |
| 50S ribosomal protein L7/L12 | *Ruminococcus*  *gnavus* | 111 (278) | 2e-028 | 58 |
| 50S ribosomal protein L7/L12 | *Ruminococcus*  *lactaris* | 111 (277) | 2e-028 | 58 |
| 50S ribosomal protein L7/L12 | *Intestinibacter*  *bartlettii* | 111 (277) | 2e-028 | 54 |
| 50S ribosomal protein L7/L12 | *Clostridium*  *bolteae* | 111 (277) | 2e-028 | 58 |
| 50S ribosomal protein L7/L12 | *Tyzzerella*  *nexilis* | 110 (274) | 6e-028 | 58 |
| 50S ribosomal protein L7/L12 | *Roseburia*  *intestinalis* | 109 (273) | 9e-028 | 58 |
| 50S ribosomal protein L7/L12 | *Mitsuokella*  *multacida* | 108 (271) | 1e-027 | 61 |
| 50S ribosomal protein L7/L12 | *Coprococcus*  *Comes* | 108 (271) | 2e-027 | 58 |
| 50S ribosomal protein L7/L12 | *Ruminococcus*  *torques* | 108 (269) | 3e-027 | 57 |
| 50S ribosomal protein L7/L12 | *Anaerotruncus*  *colihominis* | 106 (264) | 2e-026 | 52 |
| 50S ribosomal protein L7/L12 | *Blautia*  *hydrogenotrophica* | 106 (264) | 2e-026 | 60 |
| 50S ribosomal protein L7/L12 | *Clostridium*  *symbiosum* | 105 (263) | 2e-026 | 58 |
| 50S ribosomal protein L7/L12 | *Dorea*  *Formicigenerans* | 106 (264) | 2e-026 | 59 |
| 50S ribosomal protein L7/L12 | *Clostridium*  *asparagiforme* | 105 (261) | 4e-026 | 57 |
| 50S ribosomal protein L7/L12 | *Actinomyces*  *Odontolyticus* | 103 (258) | 1e-025 | 64 |
| 50S ribosomal protein L7/L12 | *Dorea*  *Longicatena* | 103 (257) | 2e-025 | 57 |
| 50S ribosomal protein L7/L12 | *Clostridium*  *sp.* | 103 (257) | 2e-025 | 58 |
| 50S ribosomal protein L7/L12 | *Clostridium*  *Scindens* | 103 (256) | 2e-025 | 58 |
| 50S ribosomal protein L7/L12 | *Clostridium*  *Sporogenes* | 102 (253) | 5e-025 | 59 |
| 50S ribosomal protein L7/L12 | *Ruminococcus*  *Obeum* | 101 (252) | 7e-025 | 59 |
| 50S ribosomal protein L7/L12 | *Clostridium*  *sp.* | 101 (252) | 8e-025 | 54 |
| 50S ribosomal protein L7/L12 | *Clostridium*  *leptum* | 101 (252) | 9e-025 | 63 |
| 50S ribosomal protein L7/L12 | *Eubacterium*  *hallii* | 101 (251) | 9e-025 | 55 |
| 50S ribosomal protein L7/L12 | *Clostridium*  *Methylpentosum* | 99 (245) | 5e-024 | 53 |
| 50S ribosomal protein L7/L12 | *Coprococcus*  *Eutactus* | 99.8 (247) | 6e-024 | 55 |
| 50S ribosomal protein L7/L12 | *Anaerostipes*  *caccae* | 99 (245) | 6e-024 | 55 |
| 50S ribosomal protein L7/L12 | *Parvimonas*  *micra* | 98.2 (243) | 1e-023 | 51 |
| 50S ribosomal protein L7/L12 | *Faecalibacterium*  *Prausnitzii* | 95.9 (237) | 8e-023 | 52 |
| 50S ribosomal protein L7/L12 | *Clostridium*  *sp.* | 94 (232) | 3e-022 | 53 |
| 50S ribosomal protein L7/L12 | *Anaerofustis*  *stercorihominis* | 94 (232) | 4e-022 | 45 |
| 50S ribosomal protein L7/L12 | *Streptococcus*  *Infantarius* | 93.2 (230) | 6e-022 | 58 |
| 50S ribosomal protein L7/L12 | *Lactobacillus*  *Salivarius* | 92.4 (228) | 1e-021 | 52 |
| 50S ribosomal protein L7/L12 | *Eubacterium*  *Siraeum* | 91.3 (225) | 4e-021 | 48 |
| 50S ribosomal protein L7/L12 | *Bacteroides*  *Pectinophilus* | 90.9 (224) | 4e-021 | 58 |
| 50S ribosomal protein L7/L12 | *Victivallis*  *Vadensis* | 91.3 (225) | 5e-021 | 57 |
| 50S ribosomal protein L7/L12 | *Faecalibacterium*  *prausnitzii* | 90.1 (222) | 8e-021 | 49 |
| 50S ribosomal protein L7/L12 | *Butyrivibrio*  *Crossotus* | 89.7 (221) | 1e-020 | 55 |
| 50S ribosomal protein L7/L12 | *Eubacterium*  *Ventriosum* | 90.1 (222) | 1e-020 | 52 |
| 50S ribosomal protein L7/L12 | *Akkermansia*  *muciniphila* | 85.9 (211) | 3e-019 | 52 |
| 50S ribosomal protein L7/L12 | *Clostridium*  *Spiroforme* | 85.5 (210) | 4e-019 | 54 |
| 50S ribosomal protein L7/L12 | *Catenibacterium*  *mitsuokai* | 83.6 (205) | 2e-018 | 49 |
| 50S ribosomal protein L7/L12 | *Borrelia*  *burgdorferi* | 83.6 (205) | 2e-018 | 47 |
| 50S ribosomal protein L7/L12 | *Erysipelatoclostridium*  *ramosum* | 83.2 (204) | 2e-018 | 53 |
| 50S ribosomal protein L7/L12 | *Providencia*  *rustigianii* | 83.2 (204) | 2e-018 | 46 |
| 50S ribosomal protein L7/L12 | *Ruminococcus*  *callidus* | 82.8 (203) | 4e-018 | 42 |
| 50S ribosomal protein L7/L12 | *Providencia*  *rettgeri* | 79.7 (195) | 3e-017 | 46 |
| 50S ribosomal protein L7/L12 | *Providencia*  *alcalifaciens* | 79.3 (194) | 5e-017 | 46 |
| 50S ribosomal protein L7/L12 | *Eubacterium*  *dolichum* | 79.3 (194) | 6e-017 | 49 |
| 50S ribosomal protein L7/L12 | *Enterobacter*  *cancerogenus* | 78.6 (192) | 9e-017 | 49 |
| 50S ribosomal protein L7/L12 | *Photorhabdus*  *luminescens* | 78.6 (192) | 1e-016 | 46 |
| 50S ribosomal protein L7/L12 | *Parabacteroides*  *merdae* | 77.8 (190) | 2e-016 | 45 |
| 50S ribosomal protein L7/L12 | *Parabacteroides*  *johnsonii* | 77.8 (190) | 2e-016 | 45 |
| 50S ribosomal protein L7/L12 | *Bacteroides*  *cellulosilyticus* | 75.5 (184) | 1e-015 | 45 |
| 50S ribosomal protein L7/L12 | *Bacteroides*  *uniformis* | 75.1 (183) | 2e-015 | 44 |
| 50S ribosomal protein L7/L12 | *Bacteroides*  *Stercoris* | 75.1 (183) | 2e-015 | 44 |
| 50S ribosomal protein L7/L12 | *Bacteroides*  *eggerthii* | 75.1 (183) | 2e-015 | 44 |
| 50S ribosomal protein L7/L12 | *Bacteroides*  *plebeius* | 75.1 (183) | 2e-015 | 46 |
| 50S ribosomal protein L7/L12 | *Bacteroides*  *ovatus* | 75.1 (183) | 2e-015 | 45 |
| 50S ribosomal protein L7/L12 | *Bacteroides*  *finegoldii* | 75.1 (183) | 2e-015 | 45 |
| 50S ribosomal protein L7/L12 | *Bacteroides*  *dorei* | 74.3 (181) | 4e-015 | 44 |
| 50S ribosomal protein L7/L12 | *Bacteroides*  *intestinalis* | 73.9 (180) | 5e-015 | 44 |
| 50S ribosomal protein L7/L12 | *Alistipes*  *putredinis* | 73.6 (179) | 6e-015 | 58 |
| 50S ribosomal protein L7/L12 | *Bacteroides*  *coprocola* | 73.2 (178) | 8e-015 | 45 |
| 50S ribosomal protein L7/L12 | *Prevotella*  *copri* | 73.2 (178) | 8e-015 | 55 |
| 50S ribosomal protein L7/L12 | *Subdoligranulum*  *variabile* | 72 (175) | 2e-014 | 44 |
| Putative uncharacterized protein | *Bacteroides*  *dorei* | 33.9 (76) | 1.4 | 24 |
| Uncharacterized protein | *Tyzzerella*  *nexilis* | 30 (66) | 4.6 | 30 |
| Putative uncharacterized protein | *Faecalibacterium*  *prausnitzii* | 32.3 (72) | 4.8 | 26 |
| Putative uncharacterized protein | *Bacteroides*  *eggerthii* | 32.3 (72) | 5.0 | 29 |
| Putative phage terminase, large subunit | *Faecalibacterium prausnitzii* | 32 (71) | 5.5 | 27 |
| Ribosomal RNA small subunit methyltransferase | *Eubacterium*  *ventriosum* | 31.6 (70) | 9.0 | 30 |

**TABLE 3.** Predicted Interferon-γ (IFN-γ) inducing epitopes within the multi-epitope protein.

| **Start-END** | **Sequence** | **Method** | **Score** |
| --- | --- | --- | --- |
| 433-448 | HIFSAAYHLSDAVQL | MERCI | 2 |
| 434-449 | IFSAAYHLSDAVQLY | MERCI | 2 |
| 435-450 | FSAAYHLSDAVQLYN | MERCI | 2 |
| 436-451 | SAAYHLSDAVQLYNF | MERCI | 2 |
| 437-452 | AAYHLSDAVQLYNFR | MERCI | 2 |
| 49-64 | EETFEVTAAAPVAVA | MERCI | 2 |
| 87-102 | LEAAGDKKIGVIKVV | MERCI | 8 |
| 88-103 | EAAGDKKIGVIKVVR | MERCI | 9 |
| 89-104 | AAGDKKIGVIKVVRE | MERCI | 9 |
| 90-105 | AGDKKIGVIKVVREI | MERCI | 11 |
| 91-106 | GDKKIGVIKVVREIV | MERCI | 12 |
| 92-107 | DKKIGVIKVVREIVS | MERCI | 11 |
| 93-108 | KKIGVIKVVREIVSG | MERCI | 11 |
| 94-109 | KIGVIKVVREIVSGL | MERCI | 10 |
| 95-110 | IGVIKVVREIVSGLG | MERCI | 7 |
| 96-111 | GVIKVVREIVSGLGL | MERCI | 2 |
| 97-112 | VIKVVREIVSGLGLK | MERCI | 3 |
| 98-113 | IKVVREIVSGLGLKE | MERCI | 2 |
| 99-114 | KVVREIVSGLGLKEA | MERCI | 2 |
| 100-115 | VVREIVSGLGLKEAK | MERCI | 2 |
| 101-116 | VREIVSGLGLKEAKD | MERCI | 2 |
| 102-117 | REIVSGLGLKEAKDL | MERCI | 2 |
| 134-149 | DEAKAKLEAAGATVT | MERCI | 2 |
| 135-150 | EAKAKLEAAGATVTV | MERCI | 2 |
| 136-151 | AKAKLEAAGATVTVK | MERCI | 2 |
| 137-152 | KAKLEAAGATVTVKE | MERCI | 2 |
| 178-193 | WFIFGPGPGQSAAII | MERCI | 2 |
| 179-194 | FIFGPGPGQSAAIIA | MERCI | 3 |
| 180-195 | IFGPGPGQSAAIIAA | MERCI | 3 |
| 181-196 | FGPGPGQSAAIIAAT | MERCI | 6 |
| 182-197 | GPGPGQSAAIIAATG | MERCI | 8 |
| 183-198 | PGPGQSAAIIAATGP | MERCI | 8 |
| 184-199 | GPGQSAAIIAATGPG | MERCI | 8 |
| 185-200 | PGQSAAIIAATGPGP | MERCI | 8 |
| 186-201 | GQSAAIIAATGPGPG | MERCI | 8 |
| 187-202 | QSAAIIAATGPGPGA | MERCI | 7 |
| 188-203 | SAAIIAATGPGPGAL | MERCI | 7 |
| 189-204 | AAIIAATGPGPGALL | MERCI | 7 |
| 190-205 | AIIAATGPGPGALLI | MERCI | 2 |
| 191-206 | IIAATGPGPGALLIL | MERCI | 3 |
| 192-207 | IAATGPGPGALLILS | MERCI | 4 |
| 193-208 | AATGPGPGALLILSN | MERCI | 4 |
| 194-209 | ATGPGPGALLILSNW | MERCI | 4 |
| 195-210 | TGPGPGALLILSNWK | MERCI | 4 |
| 196-211 | GPGPGALLILSNWKG | MERCI | 4 |
| 197-212 | PGPGALLILSNWKGP | MERCI | 4 |
| 198-213 | GPGALLILSNWKGPG | MERCI | 4 |
| 199-214 | PGALLILSNWKGPGP | MERCI | 4 |
| 200-215 | GALLILSNWKGPGPG | MERCI | 2 |
| 251-266 | YAIGPGPGSGVIGAG | MERCI | 3 |
| 252-267 | AIGPGPGSGVIGAGP | MERCI | 3 |
| 253-268 | IGPGPGSGVIGAGPY | MERCI | 2 |
| 352-367 | ISPEEWFIFAQSSIL | MERCI | 2 |
| 358-373 | FIFAQSSILSCLAAY | MERCI | 2 |
| 367-382 | SCLAAYRGNQSAAII | MERCI | 2 |
| 368-383 | CLAAYRGNQSAAIIA | MERCI | 3 |
| 369-384 | LAAYRGNQSAAIIAA | MERCI | 3 |
| 370-385 | AAYRGNQSAAIIAAT | MERCI | 6 |
| 371-386 | AYRGNQSAAIIAATN | MERCI | 6 |
| 372-387 | YRGNQSAAIIAATNP | MERCI | 6 |
| 373-388 | RGNQSAAIIAATNPA | MERCI | 6 |
| 374-389 | GNQSAAIIAATNPAA | MERCI | 6 |
| 375-390 | NQSAAIIAATNPAAY | MERCI | 6 |
| 376-391 | QSAAIIAATNPAAYT | MERCI | 6 |
| 377-392 | SAAIIAATNPAAYTE | MERCI | 6 |
| 378-393 | AAIIAATNPAAYTEQ | MERCI | 6 |
| 422-437 | AYWLAPFIIMSHIFS | MERCI | 2 |
| 423-438 | YWLAPFIIMSHIFSA | MERCI | 2 |
| 424-439 | WLAPFIIMSHIFSAA | MERCI | 2 |
| 425-440 | LAPFIIMSHIFSAAY | MERCI | 2 |
| 426-441 | APFIIMSHIFSAAYH | MERCI | 2 |
| 427-442 | PFIIMSHIFSAAYHL | MERCI | 2 |
| 428-443 | FIIMSHIFSAAYHLS | MERCI | 2 |
| 149-164 | VKEAAAKAKFVAAWT | SVM | 10.702.709 |
| 150-165 | KEAAAKAKFVAAWTL | SVM | 11.490.985 |
| 151-166 | EAAAKAKFVAAWTLK | SVM | 11.240.097 |

**TABLE 4.** Chimeric**-**MHC II epitopes and their scores provided by the IFNepitope server.

| **HTL epitope** | **Method** | **Result** | **Score** |
| --- | --- | --- | --- |
|  |  |  |  |
| ISPEEWFIFAQSSILSCL | SVM | POSITIVE | 0.23055551 |
| RGNQSAAIIAATNP | SVM | POSITIVE | 0.24625724 |
| HLSDAVQLYNFRFLLFQG  AHINAFINRNWPAIVTMA | SVM  SVM | POSITIVE  POSITIVE | 0.39891059  0.39230628 |
| TEQALLILSNWKLDP | SVM | NEGATIVE | -0.26242957 |
| QLDFDYEQFTTSI | SVM | NEGATIVE | -0.57329098 |
| WLAPFIIMSHIFS | SVM | NEGATIVE | -0.18724949 |

**TABLE 5.** Models after refinement using Galaxy Refine server.

| **Model** | **GDT-HA** | **RMSD** | **MolProbity** | **Clash score** | **Poor**  **rotamers** | **Rama**  **favored** |
| --- | --- | --- | --- | --- | --- | --- |
| Initial | 1.000 | 0.000 | 2.762 | 3.8 | 12.8 | 78.6 |
| MODEL 1 | 0.9341 | 0.458 | 1.948 | 8.6 | 1.1 | 93.1 |
| MODEL 2 | 0.9230 | 0.481 | 1.954 | 9.2 | 0.0 | 92.5 |
| MODEL 3 | 0.9326 | 0.466 | 1.968 | 9.7 | 0.9 | 92.7 |
| MODEL 4 | 0.9326 | 0.461 | 1.932 | 9.0 | 0.3 | 92.9 |
| MODEL 5 | 0.9351 | 0.454 | 1.991 | 10.1 | 0.0 | 92.5 |

**TABLE 6.** Discontinuous epitopes predicted by ElliPro server.

| **Number** | **Residues** | **Number of Residues** | **Score** |
| --- | --- | --- | --- |
| 1 | E54, V55, T56, A57, A58, A59, P60, V61, A62, V63, A64, A65, A66, G67, A68, A69, P70, A71, G72, A73, A74, V75, E76, A77, A78, E79, E80, Q81, S82, E83, F84, D85, V86, I87, L88, E89, A90, A91, G92, D93, K94, K95, I96, G97, V98, I99, K100, V101, V102, R103, E104, I105, V106, S107, G108, L109, G110, L111 | 58 | 0.877 |
| 2 | K112, E113, A114, K115 | 4 | 0.847 |
| 3 | D444, A445, V446, Q447, L448, Y449, N450, F451, R452, F453, L454, L455, F456, Q457, G458, A459, A460, Y461, A462, H463, I464, N465, A466, F467, I468, N469, R470, N471, W472, P473 | 30 | 0.8 |
| 4 | V160, W163, T164, K166, A167, A168, A169, G170, G171, G172, S173, I174, S175, P176, E177, F180, A202 | 17 | 0.708 |
| 5 | E29, L30, L31, D32, A33, F34, K35, E36, M37 | 9 | 0.659 |
| 6 | G312, P313, G314, P315, G316, T317, T318, L321, A325, I339, E342, Y343, G344, A345, E346, A347, L348, E349, R350, A351, G352, I353, E356, I360, F361, A362, Q363, S364, S365, I366, L367, S368, C369, L370, A371, A372, Y373, R374, G375, N376, Q377, S378, A379, A380, I381, I382, A383, T385, N386, P387, A388, A389, Y390, T391, E392, Q393, A394, L395, L396, I397, L398, S399, N400, W401, K402, L403, D404, P405, A406, A407, Y408, Q409, L410, D411, F412, D413, E415 | 77 | 0.658 |
| 7 | L39, L40, E41, L42, F49, T52, F53, D116, L117, V118, D119, G120, A121, P122, K123, P124, L125, L126, E127, K128, V129, A130, K131, A133, A134, D135, A137, K138, L141 | 29 | 0.658 |
| 8 | Q14, I181, F182, G183, P184, G185, P186, G187, Q188, S189, A190, L203, L204, I205, L206, S207, N208 | 17 | 0.653 |
| 9 | A23, K24, L25, S26, T27, D28 | 6 | 0.627 |
| 10 | L280, L281, F282, G283, P284, G285 | 6 | 0.60 |

**TABLE 7.** Proteasomal cleavage/TAP transport/MHC class I analysis for the multi-epitope protein.

| **Allele** | **CTL epitope** | **Proteasome Score** | **TAP Score** | **MHC Score** | **Processing Score** | **Total Score** | **MHC IC50[nM]** |
| --- | --- | --- | --- | --- | --- | --- | --- |
|  |  |  |  |  |  |  |  |
| HLA-A*23:01 | ISPEEWFIF | 1.35 | 1.11 | -1.84 | 2.46 | 0.61 | 69.4 |
| HLA-A*68:02 | QSAAIIAAT | 0.91 | -0.22 | -1.68 | 0.69 | -0.99 | 48.3 |
| HLA-A*03:01 | ALLILSNWK | 0.86 | 0.27 | -1.95 | 1.13 | -0.82 | 90.1 |
| HLA-A*23:01 | DYEQFTTSI | 1.26 | 0.10 | -3.36 | 1.36 | -2.00 | 2308.4 |
| HLA-A*23:01 | APFIIMSHIF | 1.46 | 1.10 | -1.96 | 2.56 | 0.60 | 91.1 |
| HLA-A*02:06 | GVIGAGPYAI | 1.21 | 0.23 | -2.58 | 1.44 | -1.14 | 378.6 |
| HLA-A*30:02 | SGVIGAGPY | 0.96 | 1.20 | -1.92 | 2.16 | 0.24 | 82.6 |
| HLA-A*23:01 | LYNFRFLLF | 1.29 | 1.16 | -1.02 | 2.45 | 1.43 | 10.5 |
| HLA-A*02:01 | QLYNFRFLL | 1.40 | 0.53 | -0.89 | 1.93 | 1.04 | 7.7 |
| HLA-B*58:01 | HINAFINRNW | 1.43 | 0.35 | -2.23 | 1.78 | -0.45 | 170.3 |
| HLA-A*68:02 | TTAVLAAAA | 1.09 | -0.26 | -1.16 | 0.83 | -0.33 | 14.4 |
| HLA-A*68:02 | NTNPIIESII | 1.07 | 0.18 | -1.81 | 1.25 | -0.56 | 64.7 |

**TABLE 8.** The Sequence similarity between the final selected eight proteins with other *Schistosoma* species.

|  | **Species similarity** | |
| --- | --- | --- |
| **Protein Code** | ***S. japonicum*** | ***S. haematobium*** |
|  |  |  |
| Smp_127680 | 68.3% | 83.0% |
|  |  |  |
| Smp_128250 | 73.7% | 87.8% |
|  |  |  |
| Smp_145110 | 79.0% | 76.3% |
| Smp_160120 | 86.7% | 92.5% |
|  |  |  |
| Smp_175510 | 69.9% | 81.9% |
|  |  |  |
| Smp_179660 | 81.6% | 79.0% |
| Smp_205910 | 74.2% | 88.9% |
| Smp_244490 | 77.2% | 93.4% |
